# Evolvable Neuronal Paths: A Novel Basis for Information and Search in the Brain

**DOI:** 10.1371/journal.pone.0023534

**Published:** 2011-08-26

**Authors:** Chrisantha Fernando, Vera Vasas, Eörs Szathmáry, Phil Husbands

**Affiliations:** 1 Department of Informatics, University of Sussex, Brighton, United Kingdom; 2 MRC National Institute for Medical Research, London, United Kingdom; 3 Departament de Genètica i de Microbiologia, Grup de Biologia Evolutiva, Universitat Autònoma de Barcelona, Barcelona, Spain; 4 Collegium Budapest, Institute for Advanced Study, Budapest, Hungary; 5 Parmenides Centre for the Study of Thinking, Pullach/Munich, Germany; 6 Institute of Biology, Eötvös University, Budapest, Hungary; Cajal Institute, Consejo Superior de Investigaciones Científicas, Spain

## Abstract

We propose a previously unrecognized kind of informational entity in the brain that is capable of acting as the basis for unlimited hereditary variation in neuronal networks. This unit is a path of activity through a network of neurons, analogous to a path taken through a hidden Markov model. To prove in principle the capabilities of this new kind of informational substrate, we show how a population of paths can be used as the hereditary material for a neuronally implemented genetic algorithm, (the swiss-army knife of black-box optimization techniques) which we have proposed elsewhere could operate at somatic timescales in the brain. We compare this to the same genetic algorithm that uses a standard ‘genetic’ informational substrate, i.e. non-overlapping discrete genotypes, on a range of optimization problems. *A path evolution algorithm (PEA) is defined as any algorithm that implements natural selection of paths in a network substrate.* A PEA is a previously unrecognized type of natural selection that is well suited for implementation by biological neuronal networks with structural plasticity. The important similarities and differences between a standard genetic algorithm and a PEA are considered. Whilst most experiments are conducted on an abstract network model, at the conclusion of the paper a slightly more realistic neuronal implementation of a PEA is outlined based on Izhikevich spiking neurons. Finally, experimental predictions are made for the identification of such informational paths in the brain.

## Introduction

**Figure 1:**
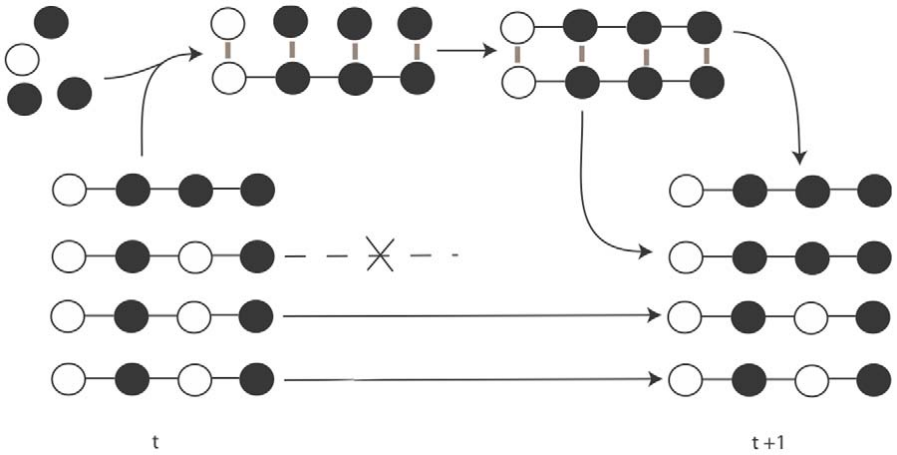
One generation of natural selection by template replication. At time t the population consists of 4 individuals with two phenotypes *b_1_* = 0111 and *b_2_* = 0101. The frequency of these phenotypes is *q_1_* = 1 and *q_1_* = 3. One generation involves template replication (possibly with mutation not shown) and removal of individuals to maintain the same population size. In the above diagram, this results in the same two phenotypes but with different frequencies *q_1_ = 2* and *q_2_* = 2 respectively. According to the Price equation, the fact that phenotypic traits covary with fitness causes fitter traits to increase in the population [5].

A unit of evolution as defined by John Maynard Smith is any entity that has multiplication, variation and heredity [1]. If units have differential fitness they can evolve by natural selection. Units of evolution [1] at the same level of selection [2] are generally considered to be discrete non-overlapping individuals, for example, living organisms, B-cells undergoing somatic selection, ribozymes in the RNA world, and binary strings in a genetic algorithm. The mechanism by which the above units multiply with unlimited heredity depends on template replication [3]. The fundamental process of natural selection using explicit multiplication by template replication to copy information is shown in Figure 1.

It was template-replication-based natural selection that inspired John Holland to invent the now famous genetic algorithm [4]. But as we will show, a discrete non-overlapping symbolic sequence, e.g. a ‘genetic’ substrate is not the only kind of unlimited heredity substrate that can be a kind of unit of evolution.

This paper proposes *an alternative informational substrate (and unit of evolution in a weaker sense) that can accumulate adaptations when in the context of a population of such units, by natural selection, but in the absence of explicit multiplication by template replication of such units.* What is more, we propose a realistic physical implementation of these units, which from now on are referred to simply as paths.

The notion of a path as a unit of evolution rests on our insight that natural selection need not act between physically independent individuals as shown in Figure 1. Instead, natural selection can act on paths in a directed graph, e.g. in a neuronal network, if the covariance between the phenotype of that path and the fitness of that path is not outweighed by transmission bias due to mutational exploration, and environmental change [5]. This more general formulation of natural selection was originally discovered by Price, i.e. natural selection takes place when there is covariance between a trait and the probability of transmission of that trait, irrespective of whether that transmission is achieved by explicitly multiplying entities as required by John Maynard Smith or by some other recipe (such as path evolution in which there is no explicit multiplication of paths). Similar ideas have been presented by Steven Frank who uses the generality of Price's formulation to describe Darwinian processes occurring in development and learning [6], [7]. George Price describes what it is to be a “natural selection cake”. John Maynard Smith describes one way to make the “natural selection cake”. Path evolution is best seen as yet another way to make this class of cake.

Paths in a network have some benefits compared with non-overlapping genetic units of evolution. The number of possible paths in a network can be far greater than the number of nodes or edges in the network because each node and edge can be part of many paths [8]. The number of possible paths in a brain-sized network is beyond astronomical; a desirable feature for an informational substrate. The same supra-astronomical property has been described for the more complex organizations known as polychronous groups (stereotyped neuronal spike patterns) observed by Eugene Izhikevich in recent models of spiking neuronal networks with delays [8]. We consider the important relation of neuronal paths to polychronous groups in the Discussion.

The majority of this paper examines a special kind of path evolution algorithm, based on a tournament selection genetic algorithm, to show the capacity for paths to act as unlimited heredity informational substrates. Having convinced ourselves that paths in networks (that have some general properties) can indeed exhibit all the crucial behaviours of a unit of evolution, we produce a more realistic neuronal path evolution algorithm based on a spiking neural network with synaptic weights modulated by Dopaminergic reward that preserves these required properties and so also allows natural selection of paths.

Using the first abstract model of paths, a standard and extremely unsophisticated genetic algorithm called a microbial genetic algorithm [9] is used to evolve paths in a network, in order to merely demonstrate that paths can in fact act as unlimited hereditary substrates for an evolutionary algorithm. From the population perspective, each path is interpreted as an individual candidate solution, one network consisting of many potentially overlapping paths/candidate solutions. Given appropriate path traversal, weight change and structural plasticity rules (that we will describe in due course) a path may be seen as a unit of evolution in the sense that it exhibits multiplicative *growth* (although not explicit replication), variation, and heredity. Each path phenotype is associated with a reward that determines whether the edges of that path are strengthened or weakened following traversal. A pair-wise tournament selection genetic algorithm (microbial GA) compares the reward obtained by two paths. The directed edges of the winning path are strengthened, whilst the directed edges of the losing path are weakened. Edges shared by both paths are not changed. Each time a node is activated there is a probability that it will mutate, i.e. produce an alternative route that bypasses that node. This generates the potential for a novel but correlated path with a novel but correlated phenotype. By this process the more frequently traversed paths are responsible for most of the exploration. Nodes that are inactive for some period of time become disconnected.

We find that the path-based GA (PEA) compares favourably with the standard gene-based GA on a range of combinatorial optimization problems and continuous parametric optimization problems. However, there are important and interesting differences. For example, the PEA more readily appears to sustain a memory of past selective environments and can store previously discovered characters for reuse in later optimization tasks. Finally, a more realistic neuronal PEA is presented showing for the first time how natural selection can occur *in a biologically plausible physical system* with unlimited heredity and yet without template replication.

What is this paper not? The main aim of this paper is to show that paths can be informational substrates in the brain. It is not to show that a microbial GA acting on paths in the present form is superior to other optimization algorithms in computer science. In fact, we do not believe that this version of a PEA in precisely its present form is implemented in the brain. It is presented here to allow a comparison between standard genetic and novel path based hereditary *substrates*. The paper is intended to convince the reader that competing, mutating, and crossing over of neuronal paths is a plausible substrate for heredity in the brain, that could potentially be used by a range of possible PEAs. In fact, the more realistic PEA presented at the end of this paper provides a demonstration that more neuronally plausible algorithms could be PEAs, i.e. use paths as hereditary substrates for natural selection.

To summarise, methodologically the justification for the comparison between a standard microbial GA and a path based microbial GA is to use a simple (and relatively unsophisticated) genetic algorithm (specifically one that does not require global operations such as explicit sorting of all genotypes) to demonstrate the hereditary capacity of **a new kind of informational substrate in the brain.** Of-course it may be possible to optimize path phenotypes using other kinds of PEA, even to use path-based information for other algorithms that are not PEAs. At the end of the paper a more realistic PEA is presented that is quite different from the microbial GA, but is still an example of natural selection of paths and hence can be called a PEA.

### Simple Examples of Paths in Networks

**Figure 2:**
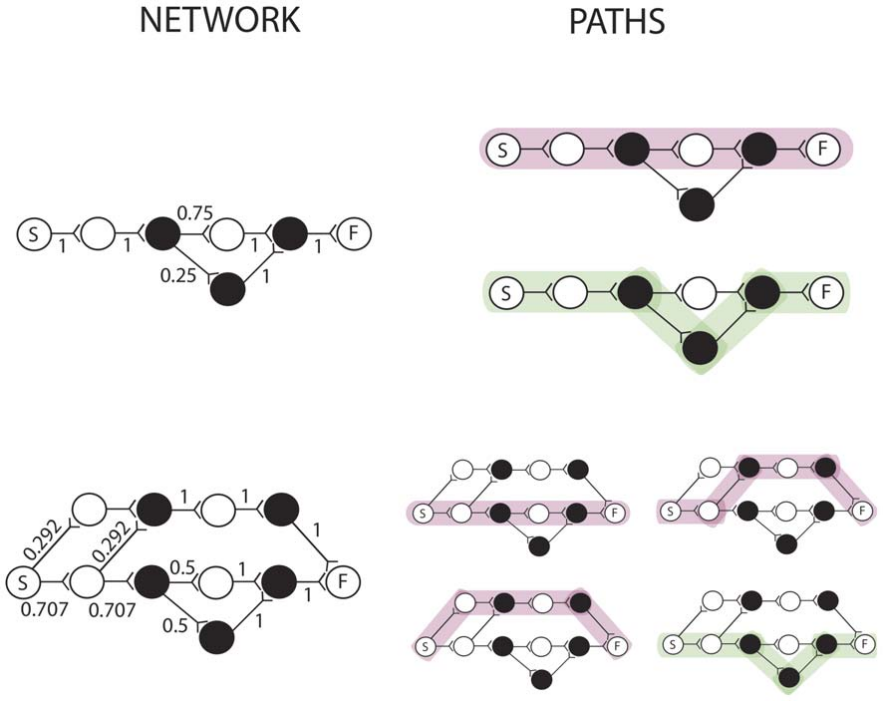
Two networks and the paths they contain. Paths with phenotype 0101 are shown in pink. Paths with phenotype 0111 are shown in green. The transition probabilities associated with each edge are marked. Note that here all the outflow transition probabilities from one node sum to one.

Figure 2 shows two networks on the left, and all the paths they contain on the right. The top network contains two paths, each of which has a distinct phenotype. The pink path has phenotype 0101 and the green path has phenotype 0111. Unfilled circles represent nodes with node phenotype 0, and filled circles represented nodes with node phenotype 1. The network on the bottom of Figure 2 contains four paths, shown on the right. Three of the paths have the same phenotype (pink, 0101) and one path has phenotype 0111 (green).

#### Path Traversal

Note that each directed edge is associated with a weight between zero and one. The sum of weighs out of one node is always normalized to one after any weight change. Weights correspond to transition probabilities (weights) *P_ij_* and are used to determine the frequency of a path. The probability of traversal (we will call this the frequency) of that path is the product of the weights *P_ij_* along that path. A node can be active or inactive. To generate a path, the start node is activated, and all other nodes are inactivated. In one time-step, the active node will then cause activation of *one* downstream node, chosen by roulette wheel selection over the outflow weights to all downstream nodes of the active node. The original active node is then inactivated. Therefore, at any one time, only one node is active in the network. This process iterates until the finish node becomes activated, at which point the path has been generated.

Given this probabilistic traversal scheme, it is easy to see that both networks at the top and bottom of Figure 2 have the same relative frequency of phenotypes as at time *t* in the traditional template based natural selection scheme shown in Figure 1. Each phenotype *b*, e.g. 0101, we will index with *i*, giving *b_i_*. Each phenotype *b_i_* has frequency *q_i_*. The frequency *q_i_* of a phenotype is defined as the sum of the frequencies of paths with that phenotype *b_i_*. The frequency of an individual path is the proportion of times that that particular path is traversed when the start node is stimulated. Note that the fact that two different networks can produce the same frequency of phenotypes (as in the top and bottom networks in Figure 2) means there is a redundant (many-to-one) encoding of phenotypes by paths, and this may permit non-trivial neutrality [10], i.e. the probability distribution of phenotypes reachable by single mutations of paths may differ depending on the underlying configuration of paths that generated them. Later we will see that this allows the network to structure exploration by learning from previous environments.

#### Paths as Units of Evolution

Whilst paths exhibit multiplicative growth, but do not explicitly replicate (multiply) in the sense that they do not reconfigure non-overlapping material to take on the same form as a parental entity. The increase or decrease of the frequency of a path occurs because there is strengthening (or weakening) of the transition probabilities *P_ij_* along a path. Whether there is strengthening or weakening of these transition probabilities depends on the reward obtained by a path. Paths are units of evolution if multiplicative growth is sufficient, rather than explicit multiplication. Note that because paths are overlapping, the multiplicative growth of one path also is multiplicative growth of parts of other paths.

Paths exhibit variation. Variation exists because each path can have a distinct path phenotype constituted by the order of node phenotypes along that path. Paths exhibit heredity by two mechanisms. Firstly, when a path undergoes multiplicative growth by increasing *P_ij_* along that path, i.e. when its frequency increases, this results in the increase of the frequency *q_i_* of its associated phenotype *b_i_* in the population of path phenotypes. Secondly, when a path mutates (to be described later) correlated variability exists because a new path phenotype, whilst not identical to the parental path phenotype, will still resemble the parent's path phenotype because a mutant path is always a short bypass of the parental path and therefore overlaps with much of the parental path, i.e. like begets like. Correlated variability was shown to be a fundamental requirement for evolvability that was lacking in a previous proposal of an alternative to template replication due to compositional inheritance [11]. Hereditary and correlated variation of paths is necessary for them to be units of evolution.

#### Node Mutations

**Figure 3:**
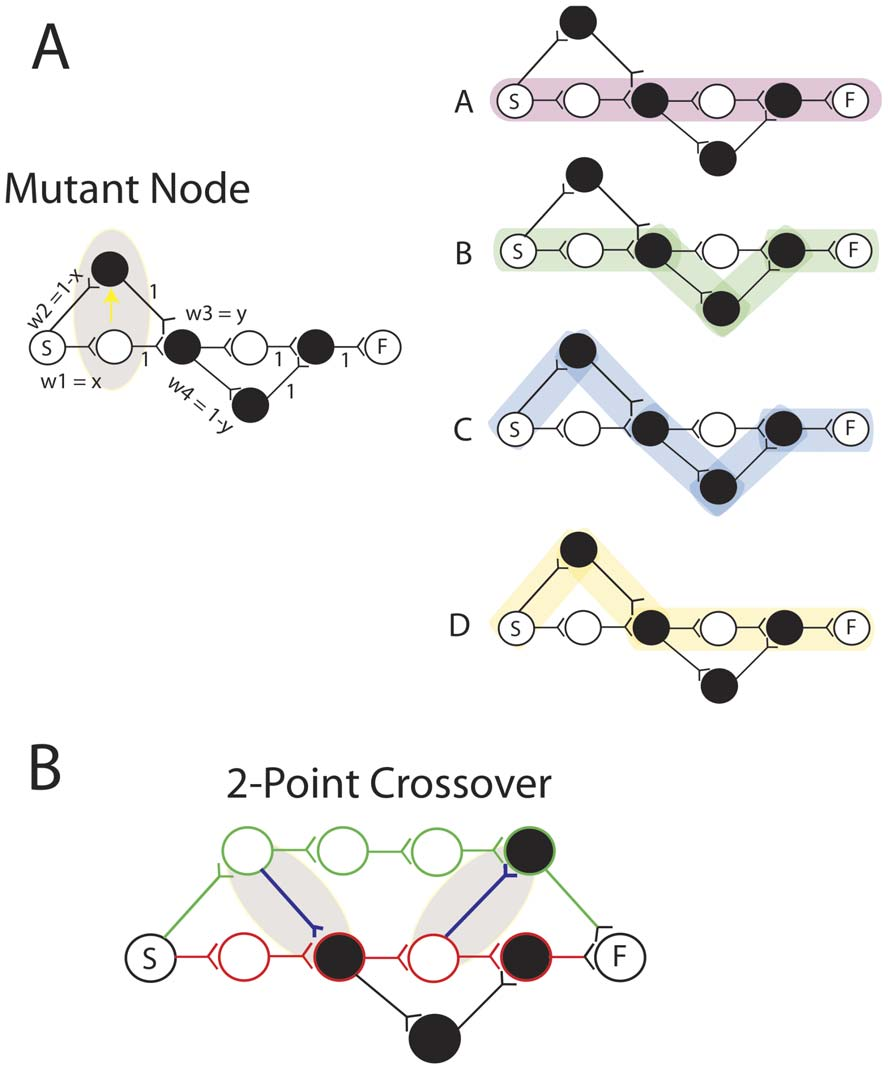
Mutation is implemented using bypass routes. (Part A) A single mutation to the network in Figure 2 produces two new paths and two new path phenotypes (Right). (Part B) 2-point crossover between a winning path (green) and a losing path (red).

The mechanism of pathway mutation is shown in Figure 3A and is based on the idea of quantal synaptic mutation originally developed by Adams [12] and for which evidence has recently been found in the form of activity dependent structural plasticity [13], [14]. On the top left is seen a mutation of the first node of the network that was previously shown at the top of Figure 2. Mutants occur with a certain probability, *μ*, each time a node is activated. A node mutation involves creating a new node at the same layer (drawn in the figures above or below the parent node). The new node has weak initial connection strength from the node that activated the parent node, and a connection of strength 1 to the node that was activated by the parent node. This preserves the original paths, yet creates new alternative paths. Note that ‘creating a new node’ can be equivalent to connecting to and from a previously existing unconnected node, and this will be the neuronal interpretation given in the later more realistic model. The path phenotypes of the alternative pathways will be correlated with the path phenotypes of the paths that contain the node that underwent a mutation. Initially the alternative paths are traversed with low probability, in other words the frequency *q_i_^m^* of a mutant path phenotype *b_i_^m^* in a population of path phenotypes will be low, if that path phenotype did not previously exist in the population. Note that this kind of mutation could not occur in the population shown in Figure 1. Because a node can be involved in many paths each having different path phenotypes, a single node mutation can change the frequency of many path phenotypes at the same time. This is one of the features that distinguish the path evolution algorithm from a standard genetic algorithm. In some cases this causes interference, but in others this allows constructive guidance of search. We will see in the simulations that the algorithm is capable of controlling the extent of overlap to suit the problem at hand, e.g. in the case where the network is evolved in variable environments, two non-overlapping paths are generated and maintained in memory.

#### Path Crossover

Path crossover occurs with probability *χ* whenever two distinct paths differ in reward, see Figure 3B. A weak weight is formed from a random layer in the loosing path to the next layer in the winning path. Another weak weight is formed from a random layer (after the first point of crossover) in the winning path to the next layer in the losing path. Thus, this is a two-point crossover that creates a new weak path that consists of part of the loosing path and part of the winning path.

### The Evolutionary Theory of Neuronal Paths

#### Evolutionary Dynamics of Paths in Fixed Networks

Let us consider the evolutionary dynamics over one generation of the simple network at the top of Figure 2. The frequency of the pink path is also the frequency of the path phenotype *b_1_ = 0101*, namely *q_1_* = 0.25, because only one path has that phenotype. The frequency of the path phenotype *b_2_ = 0111* is *q_2_* = 0.75, and is the frequency of the green path. For more complex networks the frequency of a path phenotype will be the sum over the probabilities of taking all paths with that phenotype. So now we have the frequencies of phenotypes in the ancestor generation at time *t*. Let us assume that *b_1_* has reward *r_1_ = 2* and *b_2_* has reward *r_2_ = 3*. Ignoring mutation for now, let one generation consist of choosing two paths. Each path is generated according to the roulette wheel traversal method described previously. From these two paths the winning path is chosen as the path with the highest reward associated with it. The probability of choosing path 1 twice is P(1,1) = (*0.75*)^2^. The probability of choosing path 1 and path 2 is P(1,2)+P(2,1) = 2*(0.75)0.25*. The probability of choosing path 2 twice is P(2,2) = *0.25^2^*. Only when different paths (with distinct path characters) are chosen is a winner and looser defined. Therefore, with probability 0.375 per generation, path 2 will be chosen as the winner and path 1 as the looser. The transition probabilities *P_ij_* are then modified as follows. The edges along the winning path (not shared by the losing path) will be strengthened according to the following rule…
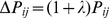
(1)and the edges along the losing path (not shared by the winning path) will be weakened according to the following rule…
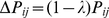
(2)for the losing path, followed by normalization over each set of outflow edges for which weights were changed. Specifically, if λ = 0.1 then the weight of the edge to path 1 will decrease from 0.75 to 0.75×0.9 and the weight on the edge to path 2 will increase by 0.25×1.1, which after normalization gives values new transition probabilities 0.71 and 0.29 respectively. By this learning rule the path character with the higher reward increases in the population and the path character with the lower reward decreases.

Let us consider a more general formulation of the above dynamics. Mathematica File S1 shows a deterministic model constructed with dynamical equations that captures the essence of natural selection in these path-based systems. A path is a genotype. A node on a path is an allele. A locus consists of all nodes on paths a certain number of nodes away from the start node (i.e. in the same layer). The frequency of a path is the probability that activity passes along that path when the start node is stimulated. The frequency of a phenotype is the probability that that phenotype will be produced when the start node is stimulated. An understanding of the system will involve a description of the dynamics and links between these various concepts.

**Figure 4:**
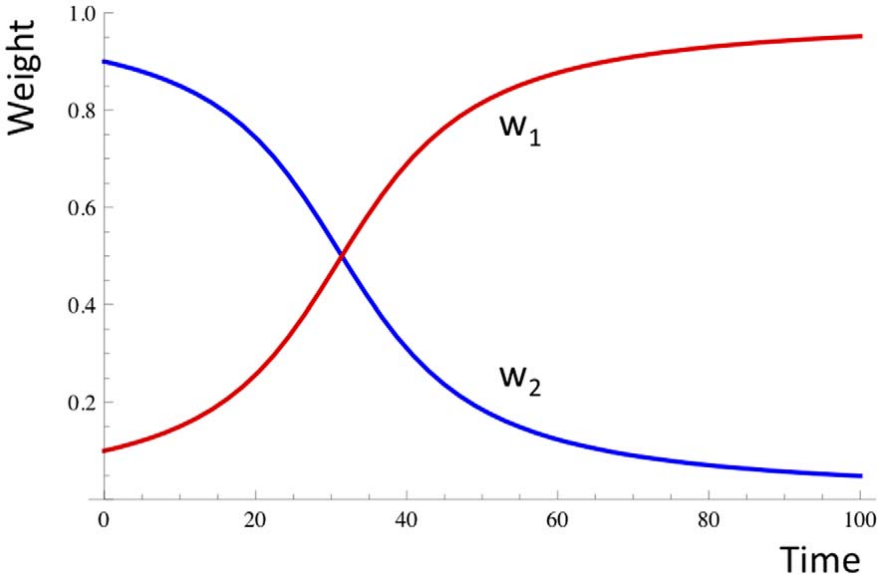
Selection between two alleles at one locus. The allele associated with higher reward reaches fixation, whilst the other allele goes extinct.

The kind of network at the top of Figure 2 can be considered as a system with one locus and two alleles. The two alleles are the two parallel nodes at the same locus (layer) of each path. Let us set the initial weight to one of these nodes as *w_1_* and the other weight *w_2_ = 1−w_1_* because the total outflow weight from the common preceding node must sum to 1. Weight change only occurs if two different paths are chosen in the two traversals available in each generation. Therefore, weight change occurs with probability *2w_1_(1−w_1_)*. With probability 1−*2w_1_(1−w_1_)* there is no weight change. Let us assume (without loss of generality) that the winning path (i.e. the path with higher reward) is associated with the node with weight *w_1_*. Then the new weight at time t+1 of *w_1_* is given by
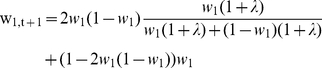
(3)For initial values w_1_ = 0.1, w_2_ = 0.9, and λ = 0.1, this gives the dynamics shown in Figure 4.

The path (and phenotype) associated with higher reward reaches fixation, whilst the one with the lower reward goes extinct.

**Figure 5:**
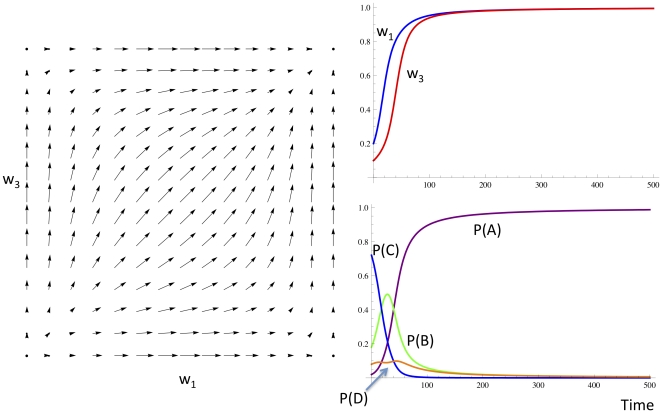
Selection at two loci, each locus having two alleles. The two fitter alleles (with weights w_1_ and w_3_) reach fixation whilst the other alleles (w_2_ and w_4_) go extinct.

Now let us consider the more complex network in Figure 3A. Here there are four paths and four phenotypes, or two loci with two alleles at each locus. Let the two weights at the first locus be w_1_ = x and w_2_ = (1−x) and we two weights at the second locus be w_3_ = y and w_4_ = 1−y. The frequency of each path is then…
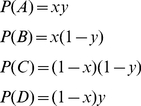
(4)Again, the weights associated with the winning path are changed as in (1) and the weights associated with the losing path as in (2) followed by normalization. Consider the cases in which w_1_ and w_2_ will change. This happens only when the path pairs AC, AD and BC are traversed with probabilities *P(AC) = 2 P(A) P(C)*, *P(AD) = 2 P(A) P(D)* and *P(BC) = 2 P(B) P(C)*, respectively. When the other pairs are traversed, either fitness is identical and there is no change in weights, e.g. (B & D), or the paths do not differ at the w_1_ and w_2_ edge, e.g when paths (D&C) or (A&B) are taken. Assume that in this case we wish to minimize the number of 1's (filled circles) in each path. Looking at each case in turn then, A beats C, A beats D, and B beats C, and so w_1_ will always be strengthened or not changed at all in each generation according to the following equation…
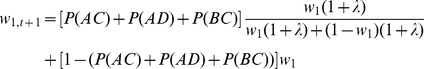
(5)Note that *w_2_* is just *1−w_1_*. Similarly, w_3_ an w_4_ will only change when path pairs AB, AC, and CD are traversed in a generation. Figure 5 shows the vector field of the Δw_1_ and Δw_3_ for the various possible values of w_1_ and w_3_, and the dynamics of allele frequencies and phenotype frequencies over time for initial conditions w_1_ = 0.2, and w_3_ = 0.1, and λ = 0.1.

The fittest alleles (w_1_ and w_3_) and the fittest path, A, go to fixation, whilst the other alleles and paths go extinct. As the vector field shows this is inevitable from any initial condition of w_1_ and w_3_. Effectively the two alleles are in linkage equilibrium.

#### Linkage Disequilibrium of Alleles in Paths

**Figure 6:**
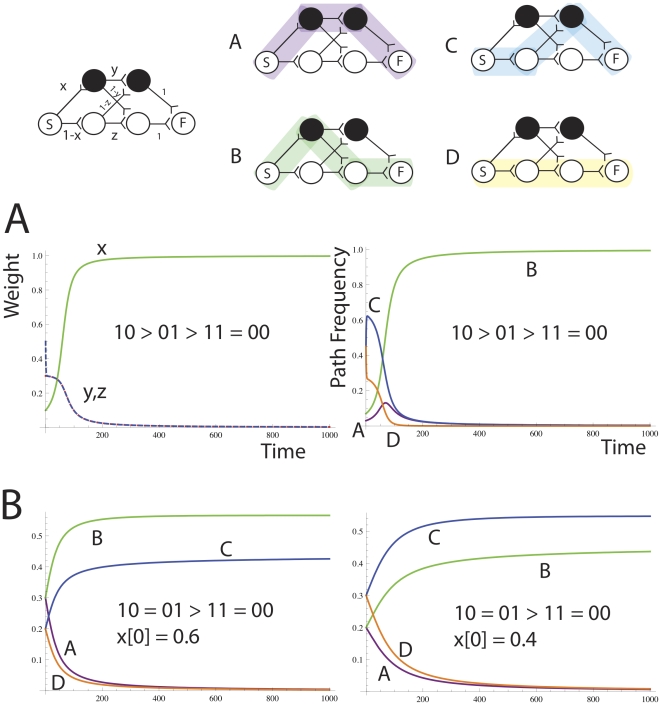
The network has three parameters x,y and z, and encodes four paths, A,B,C and D. Part A shows the dynamics of weights and path frequences for the fitness function 10>01>11 = 00. Path 10 (B, green) reaches fixation, and all other paths go extinct. Part B shows the dynamics of paths for the fitness function 10 = 01>11 = 00, for different initial weights of x of 0.6 and 0.4. Non-overlapping paths B and C are maintained at different concentrations that depend on the initial value of x. Paths A and D again go extinct.

The network in Figure 6A is initially fully connected (in the forward direction). It has two loci, each with two alleles. We show that it is possible to establish linkage disequilibrium by weight change alone. Consider the case where the ordering of reward is 10>01>11 = 00. Mathematica File S1 shows a deterministic model of how the weights *x,y* and *z* change over time to send the fittest path 10 to fixation. Alternatively, if the fitness function is 10 = 01>11 = 00, both paths 10 and 01 are maintained at non-zero probability, the ratio depending on the initial value of the weight *x*. The initially more frequent of the 10 and 01 paths reaches a higher steady state value, see Figure 6B.

The capacity to maintain non-random assortment of the alleles by i. maintaining 10 and losing 01 (in the selective case) and ii. by maintaining B and C at different frequencies in the neutral case shows the capacity for linkage disequilibrium. The network converges to make one path in the selective case, and two non-overlapping paths in the neutral case. As we saw in Figure 3A, there are some networks that will not permit the maintenance of linkage disequilibrium because it is impossible to establish two non-overlapping paths because of a node bottleneck. In this case, mutations will be required to produce a greater number of nodes at that locus, so that paths can pass without overlapping with each other, thus maintaining multiple linkage disequilibria (pairwise associations) between alleles at loci on either side of the bottleneck.

#### Assignment of Path Phenotypes

**Figure 7:**
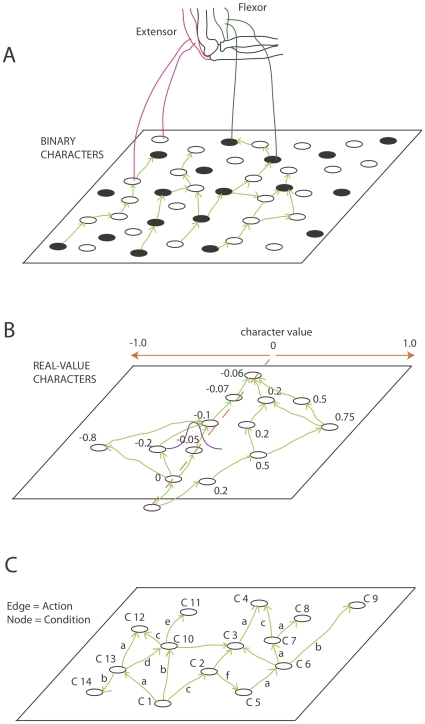
Different ways in which a path can have a phenotype. (**A**) Nodes may indirectly encode motor actions, e.g. a pattern of turns in a maze, or any other binary effector system. In this way a binary path phenotype can be encoded. (**B**) Alternatively the position of a node along the x-axis may determine a real-valued character from −1 to 1. Bypass mutants may be more likely to encompass adjacent neurons, thus producing correlated variability in phenotypes (**C**) An even more complex phenotypic interpretation of a path is to think of the network as an (anticipatory) classifier system [53] that can evolve by a modification of PE if nodes are conditions and edges are actions. A condition (t) – action – condition (t+1) triplet is a classifier.

The reward obtained by a path is a function of its phenotype *b_i_*. The assignment of a phenotype to a path is determined by how the path interfaces with the environment. This ‘environment’ may be an effector system, or another region of the neuronal network. The elucidation of realistic genotype-phenotype maps is as difficult in these systems as it is in evo-devo, however, some suggestions are given. Figure 7 shows some idealized examples of paths and their path characters and how these path characters may be associated with reward in various implementations of pathway evolution. See the Discussion for further implementation details in more realistic neuronal settings. Figure 7 part A shows that neurons may (indirectly) innervate distinct effectors such that a particular path comes to represent a sequence of motor actions, for example, at a high level in a motor system, a sequence of left and right turns may be encoded by a path. In this sense, a binary genotype can be encoded. Figure 7 part B shows that neurons may be organized into a topographic map in which adjacent positions have correlated response functions, and this is a way to encode a continuous valued genotype. Such maps are seen in early visual layers for example in which adjacent neurons have similar response characteristics. Figure 7 part C shows that a more complex kind of path phenotype may be a network of condition-action-(next condition) triplets that encodes a feed-forward model of an environment. The possibilities are in fact endless.

The full details of the PEA are given in the Methods section, and the C++ code is available in Code S1. The Results section compares the performance of PEA with various parameter settings against the equivalent standard gene based microbial genetic algorithm [9] on various combinatorial and real-value optimization problems, and for evolution in variable environments. Finally a more plausible neuronal implementation of a path evolution algorithm is presented.

## Methods

A PEA effectively maintains a rooted directed acyclic graph, with each vertex containing a reference to a parameter (phenotype) with a value for the parameter, and each edge being weighted with a normalized value, so that edge weights correspond to probabilities.

The graph is constrained so that paths are guaranteed to contain exactly one vertex for each distinct parameter. In this way, each path through the graph corresponds to a particular parameter combination, and the graph as a whole encodes a probability distribution over parameter combinations. The graph is optimized for a particular utility function by an iterative process of competitive evaluation which increases and decreases the probability of producing two stochastically generated paths, and operators which stochastically grow and shrink the graph (by adding and deleting vertices) in a manner equivalent to (dis-)connecting from previously (un-)connected nodes.

**Figure 8:**
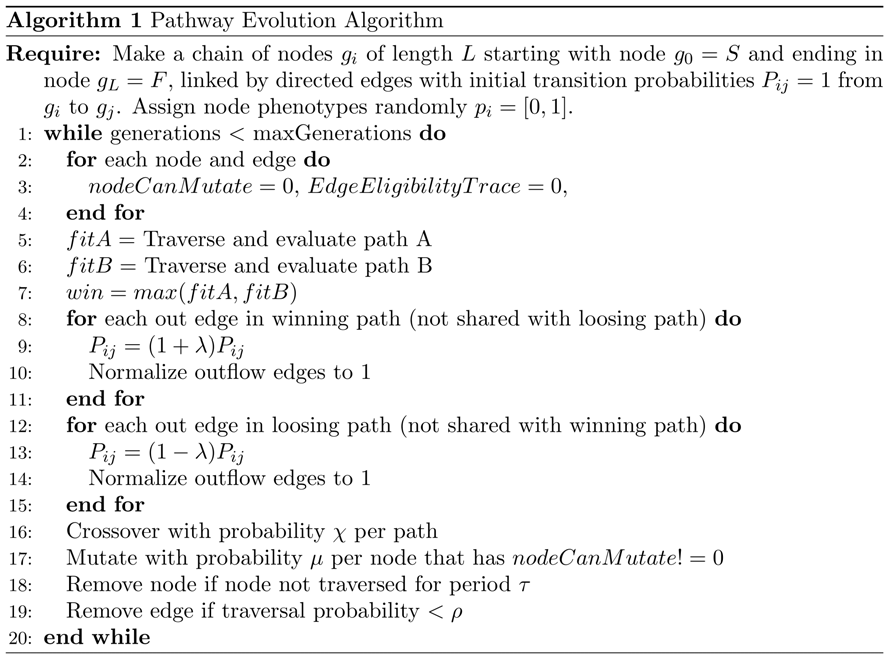
The PEA Outline. See Figure 9 for details of the path traversal, crossover and mutation functions.

**Figure 9:**
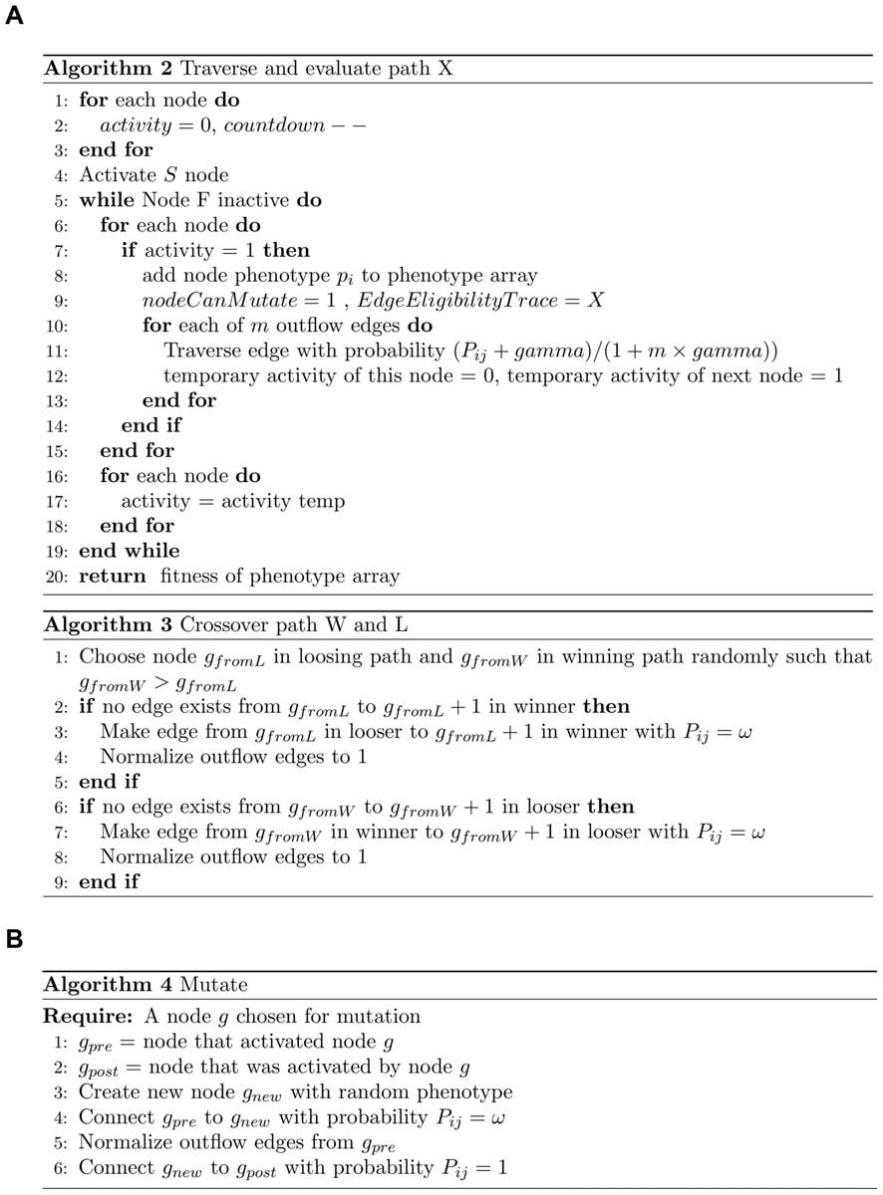
Details of path traversal, crossover and mutation operators.

The tournament selection inspired PEA is described in Figure 8 and Figure 9.

A network is initialized with *N* parallel linear directed paths (typically 1, 10 or 100 paths) of *L* nodes in length. The simplest case described above involves *N = 1*, i.e. the system starts with a single path of nodes. Let the first node be the start node that will be stimulated at the onset of each fitness evaluation. Each directed edge has associated with it a transition probability *P_ij_*. Initially all probabilities along the chain are set to 1. If the system is initialised with more than one parallel chain then the sum of probabilities out of the start node to the first node of each chain are normalized to one so that each chain is equally likely to be traversed initially. Upon stimulating the start node each node in an activated chain will fire sequentially until the end of the chain is reached. If there are many output edges from a node however, only one of the post-synaptic nodes can become active. This ensures that only one path is active at one time, and allows maintenance of path variation. A noise term can be introduced to the transition probability to promote exploration.

Because each node has a particular phenotype, each path of activity also has a phenotype. For example, if we wish to implement a binary genetic algorithm using this network then a node should be interpreted as having a label (a phenotype unrelated to the network dynamics described here) of zero or one, see Figure 4A. We randomly initialize the node phenotypes of the initial path. For example, activity passing along the initial path may produce the phenotypic sequence 0111010001. Let each phenotypic sequence so produced be associated with a reward *r*, as defined by a reward (fitness) function.

Now, at each generation, two paths are constructed by the traversal method and the reward due to each of those paths is determined on the basis of their path phenotypes. If these two paths have differential reward they compete with each other for resources. This is a tournament selection method as used in steady-state genetic algorithms. The edges along the winning path are *multiplicatively* strengthened and the edges along the losing path are multiplicatively weakened, following which all outflow edge probabilities are normalized at each node in the path. Note that if the two paths in a single tournament spatially overlap and share edges, then these particular edges are not modified. Specifically, the algorithm modifies path probabilities as follows: edge weights in favored paths are multiplied by (1+λ) (see line 9 Figure 8) and edge weights in disfavored paths are multiplied by (1−λ) (see line 13 figure 8), before renormalization. Shared edges are identified and either penalized or left unmodified, depending on the experiment. Later we investigate a diversity maintenance mechanism that involves weakening shared edges. Note that each of the two paths should have distinct eligibility traces that can be used to allocate the delayed reward appropriately.

As well as traversal probability changes modulated by reward there are structural plasticity operations occurring in the network that create and destroy edges. Node mutations can occur with a certain probability *μ* per node whenever that node is active. Mutation of a node occurs by choosing an active node *g* in a traversed path and creating a new node in that layer. The node that activated the node *g* now activates the new node, and the new node activates the node that was activated *by* the node *g*. This biases mutation to make more bypass mutant grafts around the stronger (more frequent) paths. Also it is possible to imagine the mutation operation as not one of creating a new node, but rather of co-opting an unused node from an existing node in the vicinity. Less specific variants of the mutation operator have been explored, e.g. allowing a new node to have connections from *all* nodes that were connected to its parent, or allowing it to connect to all nodes to which its parent node was connected. Generally these variability operators are more harmful. Crossover from the losing path to the winning path and back again to the losing path may occur in some runs with a low probability *χ*. This is a 2-point crossover operation that allows utilization of the useful parts of a winning path by the losing path. If a node is not active in some time period it is removed. Also, if the transition probability of an edge sinks below some threshold value, that edge it is removed.

We see that a path is a unit of evolution in the sense that it have multiplicative growth, i.e. the frequency of the path in a population of paths can increase exponentially (sigmoidally with resource limitation). There is path variation, i.e. there are many different path phenotypes maintained at one time. There is heredity, that is, a node mutation will transform existing path phenotypes into new path phenotypes that resemble the original ones (like begets like).

## Results

Several optimization benchmarks were used to characterise the PEA with different parameter settings. Performance was compared to an equivalent microbial GA but which uses a traditional genetic substrate, on binary multiple knapsack problems and on a set of parametric optimization problems. The extra ability of the PEA to show memory of past solutions was demonstrated.

### Combinatorial Optimization Problems

The binary multiple knapsack problem is an extension of the simple 0/1 knapsack problem, on which genetic algorithms have been somewhat successful [15]. A knapsack has capacity *C*, and there are *n* objects. Each object has weight *w_i_*, and a profit *p_i_*. We aim to fill the knapsack for maximum profit but without exceeding its capacity, i.e. to find a vector ***x*** *= (x_1_, x_2_…. x_n_)* where *x_i_ ∈ [0,1]*, such that 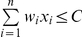 and for which 
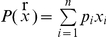
 is maximum. In the multiple knapsack problem, there are *m* knapsacks. Each object is either placed in all *m* knapsacks, or in none at all. Each of the *m* knapsacks has capacity *c_1_, c_2_…. c_m_*, and each objects has a *different* profit in each knapsack, i.e. each objects is defined by a profit vector of length *m*. Again, no knapsack must be overfilled and maximum profit must be packed.

**Figure 10:**
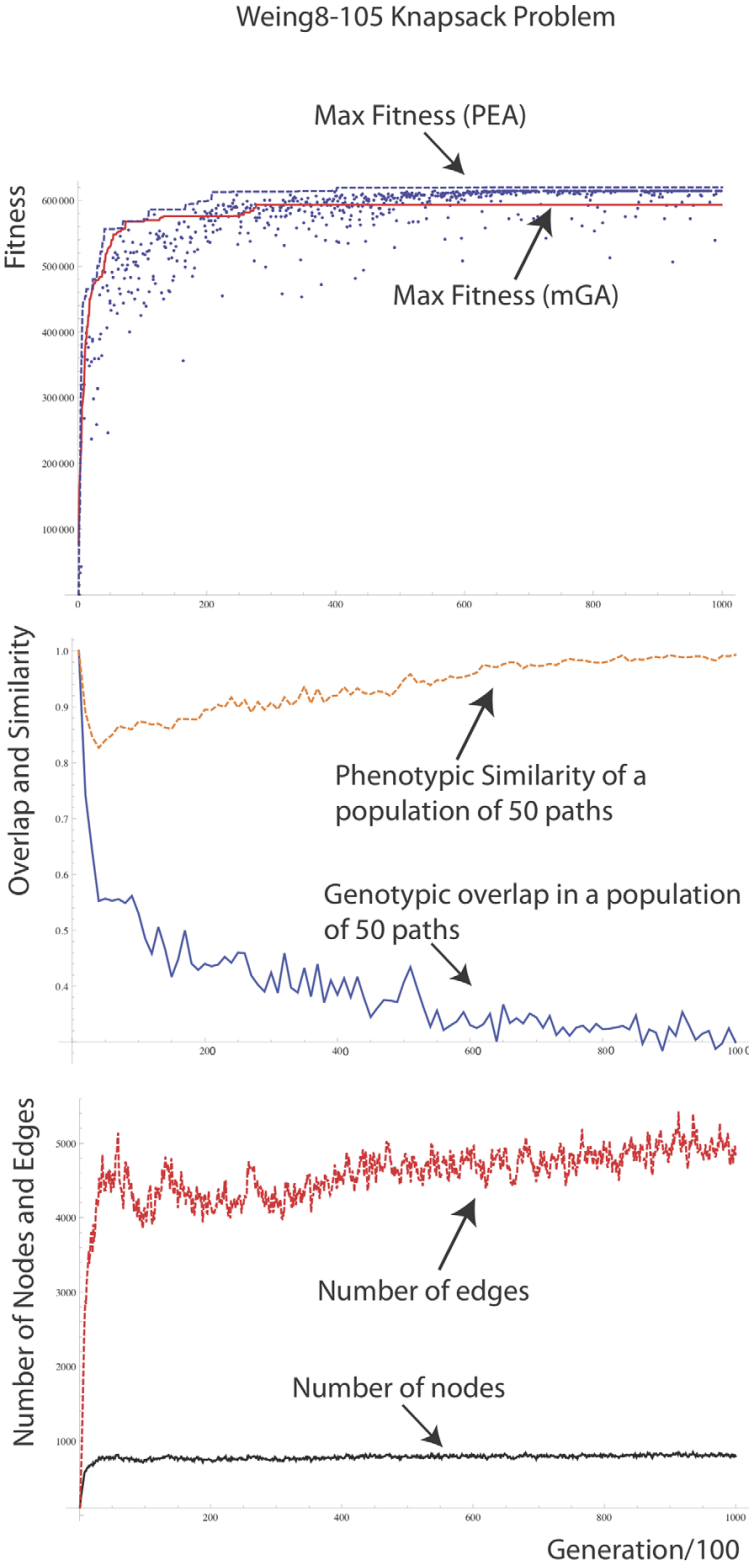
Performance of the PEA compared to a microbialGA with population size 100 and the same mutation rate on the Weing 8–105 knapsack problem. Max fitness achieved by the PEA = 620060 which is the 7^th^ best possible packing, the maximum being 624319. The following parameters were used. *N = 1, L = 105, λ = 0.1, μ = 1/L, χ = 0 (no crossover), τ = 200, ω = 0.01, ρ = 0, γ (gamma) = 0*. The PEA is run for 10000 generations, i.e. 20000 pathway fitness evaluations.

A typical run on the hard Weing8 instance of the knapsack problem [16] is shown in Figure 10. This is a hard knapsack problem with 105 objects and 2 knapsacks in which most pack vectors result in overfilling. To deal with this a punishment term is used in the fitness function that gives a negative fitness that is the extent of overfilling in all knapsacks. Otherwise the fitness is the profit of the knapsacks. On 30 independent runs, the PEA had a mean score of 615368 (sd = 7272) and the microbialGA with population size 100 had a mean score of 600236 (sd = 20003). The best solution obtained with the PEA was 622352 and the best score with the microbialGA was 623459 (the global optimum). In another knapsack problem (Weish25) the microbialGA obtained mean = 9900, sd = 40.8 and max = 9936 (the global optimum) over 30 trials, whilst the PEA obtained a mean of 9925, sd = 21.5, and max = 9936. There was no significant difference between the PEA and the GA on any of the knapsack problems we examined.

This shows that the existence of overlapping paths does not destroy the ability of a path-based microbial GA to evolve solutions to a hard optimization problem.

### Continuous Parametric Optimization Functions

**Figure 11:**
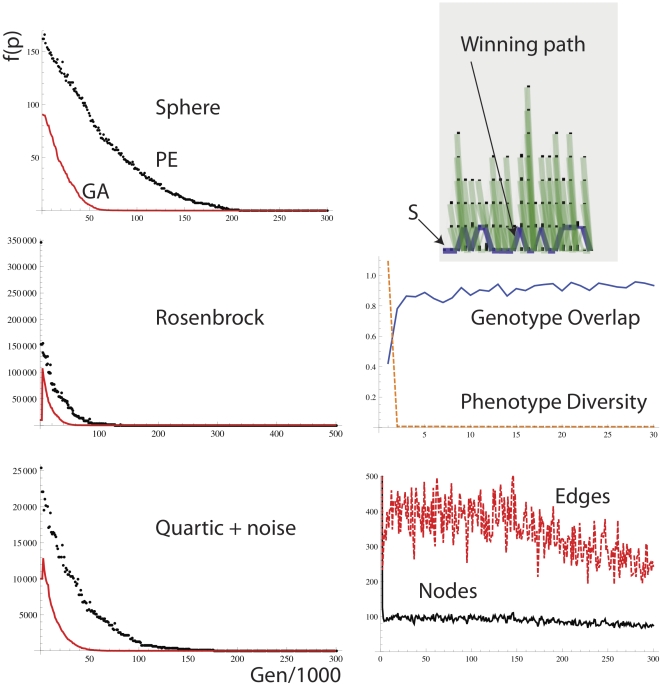
(Top) Performance of the PE algorithm on Sphere, Rosenbrock, and Quartic with noise functions. The following parameters were used: *N = 100, L = 20, λ = 0.1, μ = 1/L, χ = 0.01, τ = 100, ω = 0.01, ρ = 0, γ (gamma) = 0*. The performance details on the right are for the Rosenbrock function.

A continuous value encoding of phenotype can be defined straightforwardly as in Figure 7B. Each node is associated with a real-value number character. Mutation involves the production of a bypass mutant to a nearby node chosen as a Gaussian function (mean centered on the parental value, s.d = 0.1) of distance from the parent node. The position along the x-axis determines the real number encoded by a node. Figure 11 shows performance on the Sphere (Eq. 6), Rosenbrock valley (Eq. 7) and Quartic with noise (Eq. 8) functions, the equations for which are shown below…
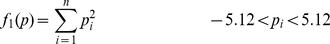
(6)

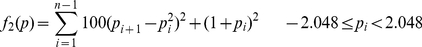
(7)

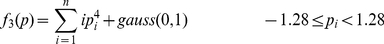
(8)Note that as opposed to the knapsack problem the function value must be minimized rather than maximized and so line 7 of Figure 8 is modified to read *win = min(fitA, fitB)*. In all runs we examined, the standard GA converges faster than the equivalent PEA to the solution. Note that the PEA with these settings behaves very much like a stochastic hill-climber (SHC), i.e. there is relatively little diversity of paths, and large path overlap between paths, see Figure 11. The number of simultaneously maintained phenotypes is low.

This highlights a fascinating feature of the PEA; it is able to modify the effective population size of paths by expanding and contracting in response to the task conditions. Sometimes it behaves like a stochastic hill-climber (SHC) with just 2 members in the population of virtual paths of activity, and at other times it behaves like a full evolutionary algorithm with a larger effective population size of paths. These effects are explored later.

**Figure 12:**
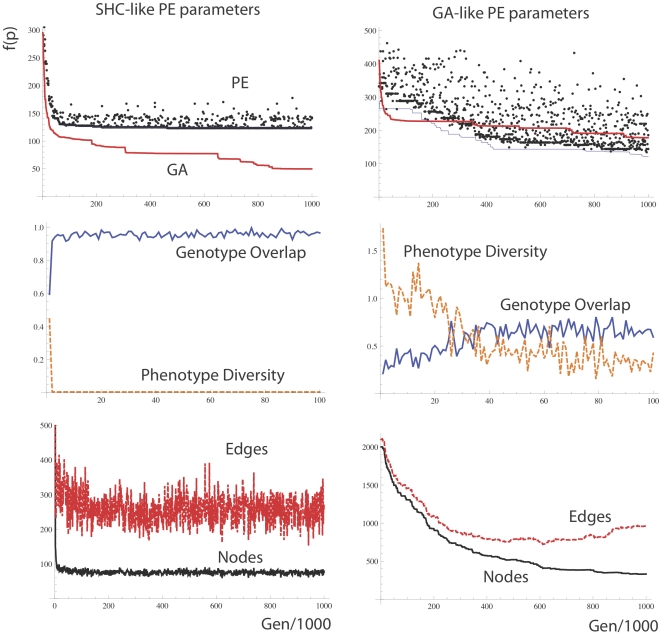
Performance of the PE algorithm on the Rastrigin function. (**Left**) PE with parameter settings as in Figure 6. (**Right**) PE with parameter settings as follows. *N = 100, L = 20, λ = 0.1, μ = 1/1000 L, χ = 0.01, τ = 1000, ω = 0.01, ρ = 0, γ (gamma) = 0.01*+overlapping edges punished as losing path. Diversity maintenance is far greater with the GA like settings that preserve distinct phenotypic niches for a longer period of time.

Interestingly, we found that with the SHC like parameter settings for the PEA, the PEA performed very poorly on Rastrigin's function which is a cosine modulation of de Jong's Sphere function used previously, see Figure 12. The Rastringin function contains many local optima and is highly multimodal with regularly distributed minima locations, see Eq. 9 below…
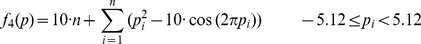
(9)However, PE with a GA-like parameter setting (Figure 12, right), in which phenotype diversity of paths is preserved for a longer period in the run, performed about as well as the GA on the Rastrigin function. To make the PEA behave more like a full population and less like a solitary search mechanism such as SHC, we increase tau (the period after which a node dies if it is not activated), we apply a non-zero *γ* for exploration during a traversal so that even after a high fitness path has been found there is still a base level of exploration. Also, we implement a diversity maintaining change to the weight modification rule in which edges that are present in both paths are in fact punished in the same way as the losing path is punished in line 13, Figure 8.

The PEA with GA like parameters performed without any significant difference to a genetic algorithm on Restrigin's function (Figure 12, right), whereas a PEA with the SHC like parameter set (Figure 12, left) always got easily stuck on a local optimum. With these GA type parameters, many more nodes and edges are maintained in the network at any one time than in the solution to the easier problems in Figure 11. Also, phenotypic diversity is slower to be lost, and genotype overlap is less throughout the run. Without punishing overlapping edges the PEA could not reach the same level of performance as the GA on this problem. This is in line with prior experience that in order to solve Restrigin's function, diversity maintenance is critical.

### Memory and Variable Environments/Tasks

Recently, as part of the extended evolutionary synthesis, people have begun to seriously study the evolution of evolvability [17], [18]. A population undergoing natural selection can automatically learn from past environments to structure exploration distributions so as to have a higher probability of producing fit phenotypes in novel but related environments [10], [19], [20]. This can occur if there is non-trivial neutrality, i.e. a many to one genotype to phenotype map in which genotypes can be discovered that produce phenotypic exploration distributions that best suit the adaptive landscape [10].

**Figure 13:**
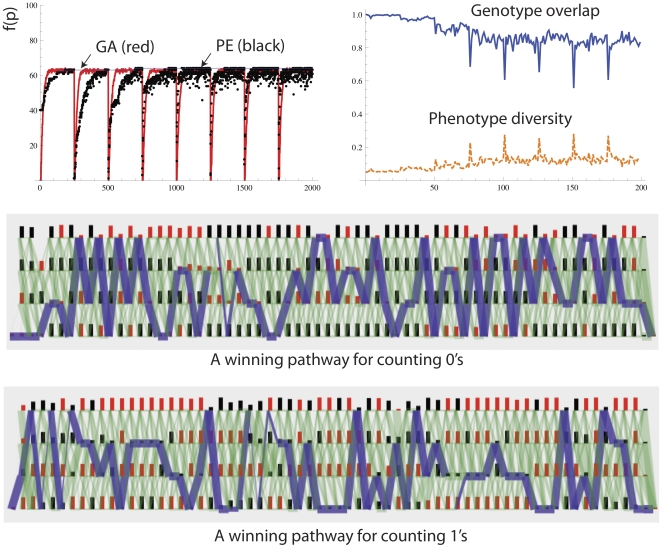
Performance of the PEA on the alternating counting 1 s and counting 0 s problem. The PE algorithm can retain memory of previously visited optima and rediscover these paths more rapidly the next time it is in the same selective environment. The GA did not improve over repeated presentations of selective scenarios. The parameters used were: *N = 100, L = 64, λ = 0.1, μ = 1/100 L, χ = 0 (no crossover), τ = 1000, ω = 0.01, ρ = 0, γ (gamma) = 0*, maximum number of nodes per layer = 4, no punishment of overlapping paths. Oscillation period = 25000 generations.

A PEA can exhibit similar automatic structuring of exploration distributions in variable environments. We present the simple example of a fitness function that involves an alternating counting ones and counting zeros problem with a period of *E = 1000* generations. Figure 13 shows that the PE algorithm is able to learn from previous environments to rediscover previously visited environment-specific optima more quickly. See that the black points more rapidly achieve optimal fitness after more and more environmental switches, but the red line (the standard GA) takes just the same amount of time to find the optimum however many environmental switches takes place. Note also that two distinct paths have been discovered for the all 1's environment and the all 0's environment.

The standard microbial GA uses a direct encoding with no capacity for non-trivial neutrality and so cannot show the evolution of evolvability, and forgets the all 1's solution once it has worked out the all 0's solution. Therefore by using this methodological comparison we have demonstrated that paths in networks have the capacity for memory of previously discovered solutions, and automatic non-trivial neutrality, which the standard population of non-overlapping genotypes lacks. The later more realistic models should lend strength to the claim that these principles should be carried over to neuroscience, and inform thinking about neuronal search.

### Problems that Benefit from Establishing Appropriate Linkage Disequilibrium: The HIFF Problem

Some problems have interdependency between variables, i.e., the fitness contribution of one variable is contingent upon the state of other variables, and there are structured dependencies that are potentially exploitable. The XOR problem considered in Figure 6 was such an example. An extension of this is the hierarchical IF-and-only-IF problem (HIFF) [21] described by the following equations…
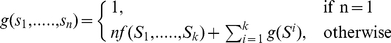
(5)Where *s_i_* is the *i* ^th^ variable of the configuration, S^i^ is the i^th^ disjoint subpartition of the variables, *f(p_1_,…,p_k_)* = 1 if 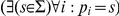, and 0 otherwise; where 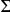 is the discrete set of allowable values for the problem variables; and *n = k^H^*, where 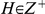 is the number of hierarchical levels in the system or subsystem, and *k* is the number of submodules per module. In HIFF we consider only binary variables, i.e., 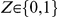 and where *k* = 2.

The lowest level of fitness contributions comes from examining adjacent loci in the phenotype and applying the transfer function and the fitness function. The transfer function is [0,0]→0, [1,1]→1, and all other pair types produce a NULL (N). The fitness function for each level just sums the 0 and 1 entries at that level. The second level is produced by applying the same transfer function to the output of the first transfer function. The fitness contribution of this next layer is again the number of 0 s and 1 s in this layer multiplied by 2. This goes on until there is only one highest-level fitness contribution. The fitness landscape arising from the HIFF problem is pathological for a hill-climber since there is a fractal landscape of local-optima, which means that the problem requires exponential time to solve. The global optima are either all 1's and all 0's.

**Figure 14:**
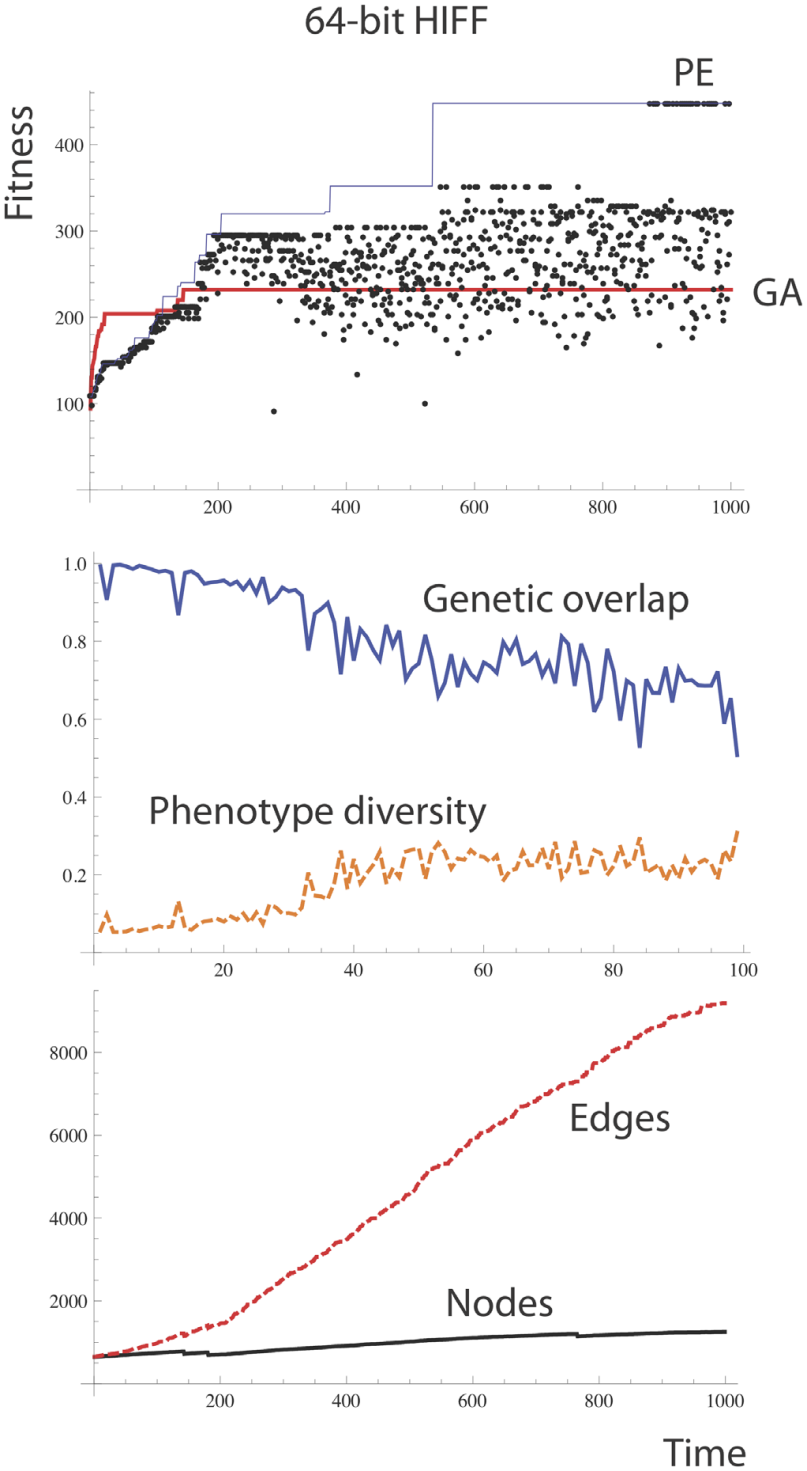
Performance of the PEA on the 64-bit HIFF Problem. The PE algorithm found the optimal solution but the microbial GA without crossover is stuck far from the optimum. The parameters used were: *N = 10, L = 64, λ = 0.1, μ = 1/100 L, χ = 0 (no crossover), τ = 10000, ω = 0.01, ρ = 0, γ (gamma) = 0*, maximum number of nodes per layer = 20, no punishment of overlapping paths.

Figure 14 shows performance of the PEA on the HIFF compared to a microbial GA without crossover using standard genotypes. PEA performs significantly better than the GA. In all 50 runs conducted, the PEA performed better than the GA. In all cases PE found the optimum by within 200000 generations, but the GA never found the optimum within this time. We propose that the good performance of the PE on the HIFF problem is because of its capacity to learn to achieve suitable linkage disequilibrium between nearby alleles. Note that poor performance on the HIFF is exhibited not only by a microbial GA without crossover, but by any GA without crossover [22], [23].

### Expansion and Contraction Dynamics during Search and Discovery

**Figure 15:**
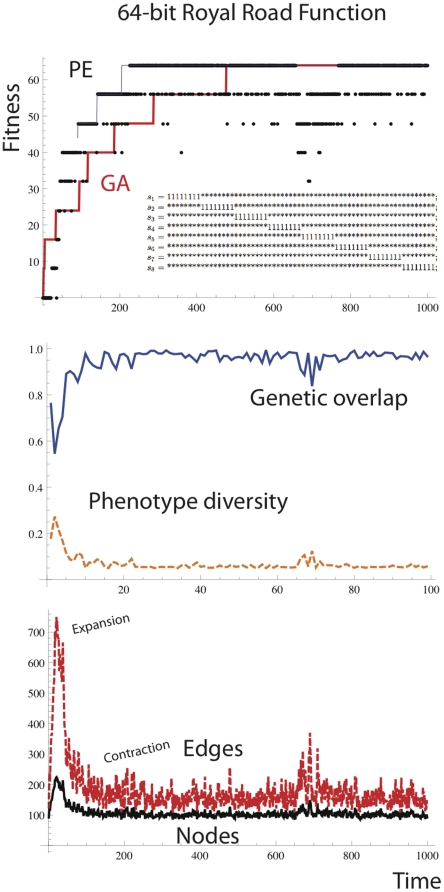
Performance of the PEA on the 64-bit royal road function shows automatic size changes. During exploration there is an expansion in the number of nodes and edges, followed by contraction after the solution is found.

Figure 15 shows the performance of PE on the royal road function. The simple royal road function is shown in the inset of Figure 15 (top).

For a bit string of length 64, 8 fitness points are obtained for each of the schemata *s_i_* that is matched by the bit string. * indicates don't care. The royal road is a royal step pyramid because it does not matter in which order the schema are accumulated. When PE is applied to the royal road, one can immediately notice that during the exploration phase there is an increase in the number of nodes corresponding to loci of the 8-bit schemata that have not yet been found. Once a schema is found, the path corresponding to that schema gains dominance, and alternative paths are lost by node deletion gradually over time. There was no significant difference between performance of the PEA and the standard GA on the royal road function over 50 independent runs.

The rather beautiful expansion and contraction dynamics exhibited by the PEA shows that there is an adaptive population size. What one cannot see here is that it is in those loci that the step has been found that the contraction takes place. So not only is there an adaptive population size, there is a locus specific adaptive population size.

## Discussion

### Is Path Evolution Really Natural Selection?

We have described the PEA using the language of natural selection: parameter combinations are ‘phenotypes’, graph modifications are ‘mutations’, increasing path probability is ‘multiplicative growth’, node parameter-values are ‘alleles’ and so on. This arises from our interest in the neuronal replicator hypothesis that considers whether evolutionary computation may be possible in the brain [24], [25], [26], [27], [28], [29]. In fact, viewing network processes from the evolutionary perspective was crucial in allowing us to see paths as possible hereditary substrates. We also have a longstanding interest in the origin of life and therefore we notice that the algorithm also shows how natural selection can occur in the absence of template replication in a physical system. Template replication was previously thought to be necessary for natural selection with unlimited heredity [3]. It is not.

A skeptic may ask, can a path of activity really legitimately be considered to be a unit of evolution? John Maynard Smith said that group selection requires the existence of cohesive, spatially discrete groups, that “reproduce” by sending out propagules, and that can go extinct (1976, p. 282). He defined a population of units of evolution as “any population of entities with the properties of multiplication (one entity can give rise to many), variation (entities are not all alike, and some kinds are more likely to survive and multiply than others), and heredity (like begets like) will evolve. A major problem for current evolutionary theory is to identify the relevant entities” (p. 222, [30]). We have identified a path as a unit of evolution, however it is not a spatially discrete physical individual in the way John Maynard Smith imagined, it has multiplicative growth rather than explicit replication. A path is capable of multiplicative growth in the population of paths; however, it does not give rise to a distinct spatially separate entity during growth, but strengthens the probability of traversal of its edges. We have demonstrated that path characters can have variation and heredity, by bypass mutations.

So the PEA (in this case, a microbial GA acting on paths) implements something that is similar to *and* different from a conventional natural selection acting on genetic informational substrates as modeled with the microbial GA acting on discrete non-overlapping genotypes. The differences are as follows…

1. Whilst there are a well-defined number of distinct paths in a physical network, e.g. 4 paths in figure 3, the relative frequency of a path in the virtual population of paths generated by repeated stimulation of the start node is a probability. In standard natural selection the frequency of a genotype is an integer value.
2. In the PEA a single mutation can affect multiple genotypes whereas in standard natural selection a single mutation can affect only one genotype. Thus, individuals are non-distinct on the evolutionary level.
3. In the PEA, multiplicative growth and selection operators will in general have direct side effects on the prevalence of many genotypes besides those that were directly evaluated under selection. Thus, individuals are non-distinct on the ecological level.
4. The PEA has memory for past environments. Paths that were useful in past environments can be more stably preserved than in the population of a standard microbial GA.
5. The PEA exhibits an automatic capacity for non-trivial neutrality because there is a many to one network to phenotype map with some mappings possessing favorable exploration distributions [10]. This was not an automatic feature of the standard microbial GA.
6. The PEA can automatically establish appropriate linkage disequilibrium by controlling the amount of overlap between paths, and is thus able to solve the HIFF problem where the microbial GA is not.

A skeptic may claim that rather than demonstrating that a “true Darwinian process” is possible in the absence of distinct units, the paper suggests that the concept of evolution by natural selection is inherently less well defined than previously assumed. If an evolving population can be an implicit one, then this significantly widens the net of processes that could be described as evolutionary. Perhaps even processes as physically simple as annealing could be given an evolutionary slant in this sense?

We disagree. Here it is helpful to consider a classification of optimization algorithms, see Table 1.

**Table 1:** A classification of search (generate-and-test) algorithms.

Solitary Search	Parallel Search	Parallel Search with Competition (Price)	Parallel Search with Competition and Information Transmission (JMS)
(Stochastic) hill climbing/Simulated Annealing	Independent hill climbers, e.g. with restart	Competitive Learning	Genetic Natural Selection
Markov Chain Monte Carlo		Reinforcement Learning	Adaptive Immune System
		Synaptic Selectionism	Genetic Algorithms
		Neural “Darwinism”	Neuronal Replicators

In solitary search only one candidate solution is maintained. Examples include hill-climbing and stochastic hill-climbing. Next, it is trivially possible to parallelize solitary search. This we call parallel solitary search, and doing so allows a linear speed up. In a denuded sense this is a population of sorts. Increasing in sophistication one may allow parallel solitary search with competition. Here there is competition for a global search resource that can be reassigned between individual candidate solutions, probably to the currently best candidate solutions, where best may have a potentially complex definition. In effect, there is now a simple ecology of competition between candidate solutions. Into this category falls competitive learning [31], [32], Hebbian learning [26], [33], many reinforcement learning algorithms (in which the competing units are state-action pairs) [34], and other action [35], [36] and attention selection [37] models. However, all these models lack information transmission *between* candidate solutions. This defines a new category of search called parallel solitary search with competition and information transfer between candidate solutions. Natural selection is the archetypical example of this class of algorithm. We call such a population a Full Population. It converts a competitive ecology into a true evolutionary system. Notice that the Price equation is satisfied even by the third class of search, and so in a sense it is a broader definition of natural selection that does not explicitly require information transmission between solutions.

Note that in a genetic natural selection, information transfer between candidate solutions occurs in a fixed population size with mutation alone (crossover is not needed). To see this is the case, imagine there are 10 material slots, each configured as a particular candidate solution. When a candidate solution replicates with mutation, a randomly chosen slot is reconfigured with a mutated configuration generated (copied) from the parent candidate solution. By observing the state of the offspring slot that was reconfigured one can reduce ones uncertainty about the parental solution. Thus there is transfer of information between material slots. The PEA contains a kind of information transfer because paths overlap, and bypasses can connect paths together that were previously unconnected.

Our algorithm shares with natural selection in organisms, and artificial selection in genetic algorithms, the following properties: a full population with information transmission between individuals and competition between individuals, unlimited heredity (the capacity for long paths/genotypes), and (for the problems considered) covariance in fitness between parent and offspring (i.e. the capacity for micro-mutation by short path bypasses/mutations). In this sense, it follows the spirit and the letter of the law of natural selection, but uses a novel hereditary substrate that adds a rather strange set of previously unnoticed novel properties.

### Related Approaches in Computer Science

There is a related set of algorithms used in computer science, specifically in evolutionary computation. For example, a class of algorithms exists called estimation of distribution algorithms (EDAs) that do not explicitly represent the individuals in a population at all, instead they maintain a probabilistic description for the probability of an allele occurring at each locus, and the novel solutions are obtained by sampling from this distribution. Whilst the path evolution algorithm may therefore be seen as a kind of EDA, it's method of updating the probability distribution of solutions is quite different from the methods traditionally used in EDAs [38]. As far as we are aware one of the goals of EDAs was to remove “arbitrary” operators such as mutation and crossover. This was not our goal in developing the path evolution algorithm in which we stress the importance of generative operators. The path evolution algorithm has no explicit re-construction of a probability distribution on the basis of only the best individuals at each generation. EDAs suffer from the problem that the estimation of such a distribution may be unreliable for a large problem size, *therefore EDAs typically make simplifying assumption that alleles at different loci are in linkage equilibrium*, in other words that the probabilities of alleles occurring at separate loci are independent variables (e.g. the univariate marginal distribution algorithm UMDA, the population based incremental learning algorithm PBIL, and the compact genetic algorithm CGA). More sophisticated approaches may consider bivariate dependencies (two locus models) or multiple dependencies. In contrast, *the path evolution algorithm can automatically explore multiple dependencies between alleles*. It does this by adapting the network structure by using simple local structural operators that could be implemented in a biologically plausible neuronal network. EDAs do not fall into the category of full population search with competition and information flow between solutions, because they exhibit no information flow between solutions, as there is in path evolution. In short the PEA provides a much simpler and more elegant framework that (as we will show) has a plausible neuronal implementation.

Ant colony optimization (ACO) algorithms were not inspired by the idea that natural selection might occur in the brain, but by the communication between ants about the best paths to food [39]. Unlike EDAs, ACOs do fall into the category of full population search with competition and information flow between solutions. However, interestingly, a recent survey states that it is still an open research question “how and why the method works” [40]. We believe that our explanation here of the function of the path evolution algorithm is the best explanation so far for how ACO like mechanisms actually work. That is to say, they work by the natural selection of paths. It is remarkable that this explanation appears nowhere in the ACO literature, however it is not entirely surprising for natural selection is often cryptic as an explanation for adaptation in systems that superficially may appear to lack it [41].

ACOs are slightly more complex than the path evolution algorithm because they determine whether a traversal is ‘feasible’ by referring to the phenotype of a node. In the PEA, phenotype “semantics” never influence genotype “syntax”, i.e. there is no “heuristic information” as in ACOs. We hope that the PEA will be welcomed by the ACO community as a general explanation for the adaptive power of ACOs.

Note that particle swarm optimization also falls into the category of full population search with competition and information flow between solutions [42]. The information exchanged (replicated) is the memory of the location in N-dimensional space of local optima between particle (slots). Particles are physical slots between which information is exchanged. Strangely, particle swarm optimization also works by a process of Darwinian natural selection in which the replicator is location information. Confusion arises when people think replication is replication of matter rather than of information. Thus, particle swarm optimization is not made Darwinian by replicating particles themselves!

The network we maintain in path evolution is a kind of hidden Markov model but with a rather restricted feed-forward topology [43]. The problems for which HMM learning algorithms are used are not optimization problems but supervised learning problems requiring generalization, in the sense that the final set of desired parameters (outputs) are known, e.g. the desired outputs in the training set may be a string of nucleotide sequences. Our problem is slightly different. We have an unknown set of optimal outputs, and we must use immediate reward information to generate a HMM for them. Viewed in this light, this paper provides an algorithm based on natural selection of paths that is able to produce hidden Markov models for optimization problems.

For traditional HMM problems, usually, heuristic algorithms such as Baum-Welch (iterative maximum likelihood estimation) [44] are used to produce a model that can generate this *known* set of desired outputs. These algorithms may get stuck on local optima because they are solitary (gradient climbing) algorithms. Also, they require assumptions about model size and topology. Previously, evolutionary approaches have been used to evolve HMMs for such problems, e.g. for protein secondary structure prediction by Rene Thomson [45]. However, these algorithms maintain multiple separate HMMs and use operators such as add state, remove state, modify state phenotype, add/delete transition, and crossover between distinct HMMs. They evolve an unlimited number of HMM topologies, including recurrent topologies. Also, they add a component of fitness that is linked to the Bayesian Information Criterion to compress the HMM [46]. In contrast our approach is an evolutionary approach for evolving a single non-recurrent HMM containing multiple paths for optimization problems without supervision, and so far, no capacity (yet) for compression guided by BIC.

### A Slightly More Realistic Neuronal Implementation of a Path Evolution Algorithm

The neuronal networks of the brain provide the most natural implementation of paths as informational substrates with unlimited heredity. We produced a model using Izhikevich spiking neuronal networks as described in [47] but with some modifications that are needed to convert the network to run a PEA that is clearly recognizable to the naked eye.

**Figure 16:**
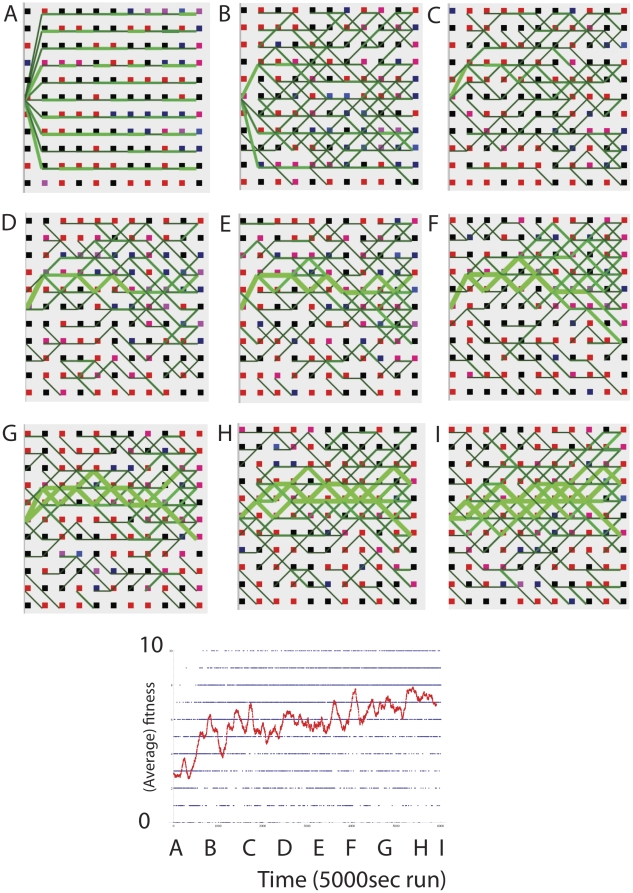
Izhikevich spiking neuronal network modified to include WTA output competition and activity dependent weight decay and plasticity, solving the 10 bit all 1's problem. Red squares = neurons with phenotype 1, Black squares = neurons with phenotype 0. Thickness (and lightness) of green lines = strength of weights from 15 mV to 60 mV (max). The figure below shows the fitness of the path phenotype in each run, and the moving average used to determine whether to give reward or not, over 5000 trials (with 1 trial per second).

Figure 16a shows the initial state of the network of regular spiking neurons that form 10 initial paths, each path being stimulated by a start neuron. The initial weights are set to a random value between 15 mV to 60 mV (maximum weight = 60 mV). This allows a path to be created by single neurons. If weights are made weaker, many neurons are required to sustain a path and the system is considerably more complex. 10 ms into each second, the start neuron is externally depolarized causing it to fire. This results in activation passing downstream activating each neuronal layer. Neurons are connected by delay lines of 1 ms, although variable delays can also be used. Background noise is set so that neurons on average fire at 0.1–1 Hz. Synapses are modified by STDP via eligibility traces modulated by DA reward, as in Izhikevich's paper [47] except that eligibility traces decay 10 times faster and reward decays 4 times faster than in the original paper, thus increasing the specificity of reward.

#### Winner-Take-All Competition at Outputs

An important modification to Izhikevich's model must be made. To implement a hidden Markov model type network it is necessary to limit the outflow of information from one node to just one possible output. In spiking neurons, this is achieved by winner take all (WTA) output competition between all outflow paths for activation. This introduces variation upon which selection can act, and ensures a single path is generated at a time (rather than a tree of spreading activation). This in turn means that only one path is responsible for behavior and hence credit can be specifically assigned to just that path. Effectively, weight proportionate WTA output competition produces a system with very sparse activation, which helps with specific credit assignment. Many neuronal models assume WTA competition, for example self-organization of spike pattern sensitivity in neurons with winner take all (WTA) lateral inhibition and STDP [31]. This competition results in the frequency distribution of single spike outputs matching the weight distribution of output synapses.

### Phenotypes in Spiking Neuronal Networks based on Spike Order

The phenotype of the network is also interpreted differently from Izhikevich [47]. At 10 ms into each second, the start node is stimulated and a sequence of spikes is produced. This sequence is of varying length, i.e. activity may not propagate all the way to the final layer. Each node is assigned a node phenotype (0, or 1) as in the previous PEA. The identities of the first 10 nodes that spike after stimulation at 10 ms is recorded in an array, and the phenotype is defined as the binary string produced by this ordered list of node phenotypes. In the counting 1 s problem, the fitness of this binary string is the number of 1 s contained in it, and this determines the dopamine reward given at 50 ms.

### Differential Growth and Selection of Pathways by Dopamine Modulated STDP and Weight Decay

Reward is also given in a different way to Izhikevich [47]. At 50 ms into each second, reward is given according to the fitness of the path phenotype compared to a running average fitness window. Running average fitness is defined as fitness average(t+1) = 0.01 x fitness average(t)+0.99 current fitness. If the current fitness (i.e. the reward obtained from the path phenotype of the spikes produced between 10 ms and 50 ms into each second) is greater than the average fitness, then reward is given at 0.5 units of DA per correct bit. The fitness function we use is simply the all 1's task, where we wish a sequence of 10 neurons each with a neuron phenotype of one to fire immediately after the 10 ms stimulation, before any neurons with the 0 phenotype fire. If the current fitness is greater is less than the average fitness then a negative reward is given at 0.5 units of DA per incorrect bit. This simple method of assigning reward is primitive when compared to a full TD learning mechanism that modifies reward up and down on the basis of a difference from the predicted reward, but it approximates this and still works. Reward then modulates weights (up and down) on the basis of their eligibility traces. The forth modification to Izhikevich is that in addition to weight change due to DA modulated eligibility traces based on STDP, there is an **activity dependent** linear **weight decay** of 0.00002 mV per ms, if a neuron does not fire at all in one second; this results in a weight reaching the minimum permitted weight of 15 mV within approximately a minute if it does not fire at all.

### Activity Dependent Structural Plasticity Implements Mutation

The fundamental operation of node mutation and pathway crossover that the PE algorithm depends upon is closely related to the synaptic pathway mutations first proposed by Adams [12] in which Hebbian learning is noisy, i.e. when a synapse is strengthened there is also a small probability that synapses will be strengthened from the pre- or the post- synaptic neuron to or from nearby neurons. Adams' insight prefigured the recent discovery of rapid structural plasticity; the formation and breakage of synapses in the order of minutes [14], [48], [49]. These operations are eminently suitable for implementation of the bypass mutations required for neuronally plausible path evolution. In real neuronal networks it is possible that path mutations will be able to shortcut several layers, or add layers, producing variable path lengths. Also, recurrent paths may come to exist. However, for purposes of demonstration here we chose to add a simple kind of **activity dependent structural plasticity** to Izhikevich's model that is constrained in the topology of connectivity that is possible by mutation.

Whenever a neuron is active there is a 1% probability that it will produce a new synapse to an adjacent neuron (i.e above, same or row below) in the *next* layer (column). The neuron to which this new output passes also produces a new output randomly to an adjacent neuron in *its* next layer, thus there is a 1/3^rd^ probability that a bypass mutant is produced, and a 2/3^rd^ probability than a divergent mutation (crossover) is produced that does not return to the original path. If a weight decreases below the minimum level of 15 mV it is removed. No neuron may have more than three output synapses. Whenever a new synapse is formed, if the total weight of synapses out of a neuron exceeds 60 mV, then one synapse is removed from the output synapses of that neuron, in inverse proportion to its weight. These activity dependent structural plasticity rules bias synaptic exploration to those neurons that are currently most active.

To overcome the limitation that the random generation of node phenotypes may produce a matrix of neurons that does not contain a single possible path of all 1's from the start node to one of the final layer nodes, we allow random node phenotype bit flipping at a low rate, e.g. once every 1 minute iff that neuron has not spiked once in this time.

Figure 16 shows an evolutionary experiment conducted with a realistic neuronal implementation of pathway evolution that uses Izhikevich spiking neurons, WTA competition, structural plasticity and dopaminergic reward to evolve pathways. This simple demonstration shows that pathway evolution can be expected to carry over to more realistic neuronal implementations using more realistic reinforcement learning kinds of reward allocation. The network of pathways can be seen as overlapping models to which reinforcement can be given [50], [51]. What is special here is that the models are evolved in a realistic spiking neuronal network. The software for running the above simulations can be downloaded from Code S1.

### Conclusions

There are several points where the spiking model is unrealistic. It would benefit from a simulation with many more neurons and weaker connections between neurons. In this case, it is likely that polychronous groups would be the primitive units (nodes) forming a path [8], [52]. In other words, the path would be a kind of trajectory in state space, rather than a localized neuronal pathway. Each state in the trajectory would consist of the activation of a polychronous group. This is a further step in abstraction that we hope to consider in later even more realistic models. However, we have used this simplified system to help us think about a preliminary mapping of natural selection onto networks of more general form. We believe this is the genuine novelty of this paper.

Also real neuronal networks are recurrent. Preliminary modeling has shown that recurrence produces several problems for the algorithm. If A causes B to fire, and B causes A to fire shortly afterwards, then due to STDP the edibility trace associated with the synapse from A to B is both strengthened and weakened in succession. Therefore, this synapse is not rewarded as much as a chain of synapses would be. Further work is needed to extend PEA to recurrent neuronal networks.

Here we have demonstrated that overlapping paths in networks can be a hereditary substrate, yet without being spatially distinct individuals. Paths are capable of evolution by natural selection. Pathway evolution has several features that distinguish it from standard genetic evolution. These all result from the fact that paths overlap. Path overlap may be good or bad, and we have shown that the extent of path overlap can itself be determined by the PEA, see Figure 13 for a clear case of this.

The capacity to implement path evolution in the brain with a relatively trivial modification of existing models, lends very strong support to the neuronal replicator hypothesis, that argues that there exist informational replicators in the brain, i.e. autocatalytic entities capable of producing offspring that are correlated with their parent in fitness, and hence capable of accumulation of adaptations by natural selection [24], [25], [26], [27]. Path evolution allows rapid search by activity distributions to modify the frequency of a solution, encoded as synaptic weights. We have not restricted ourselves to a particular cognitive architecture here, but have merely suggested a particular kind of generative variation in neuronal networks that may allow unlimited heredity of information, for a range of possible algorithms.

We hope that neuroscientists will be interested in taking the path perspective. For example, in the neurosciences one may attempt to identify paths, and observe their multiplicative and mutational dynamics. One may ask what is the probability of fixation of a novel pathway or edge (synapse) in a real neuronal network as a function of the reward it obtains?

Finally, we point out that other implementations are also possible, hence the level of description we have chosen to present the path evolution algorithm. For example, in chemical reaction networks, the internet, or social networks, it is possible that network adaptation takes place by path evolution, a kind of cryptic Darwinism. All that is required is the ability to assign reward to a path, and path growth with bypass mutations. John Maynard Smith's goal applies now as it always has; a major problem for current evolutionary theory is to identify the units of evolution. We claim that the task of identifying the units of neuroevolution is a prescient task for neuroscience, and one that we hope to have defined and contributed to here; showing that units of evolution can overlap thus allowing Darwinian natural selection to operate in a cryptic form in the brain.

## Supporting Information

Mathematica File S1 **A Mathematica file showing the analytical treatment of the single locus and double locus path evolution algorithm.**
(NB) Click here for additional data file.

Code S1 **XCode C++ files for running the PEA on the Royal Road Function.**
(TAR)

## References

[1] Maynard Smith J (1986). The problems of biology.

[2] Okasha S (2006). Evolution and the levels of selection.

[3] Szathmáry E (2006). The origin of replicators and reproducers.. Philos Trans R Soc London B Biol Sci.

[4] Holland JH (1975). Adaptation in Natural and Artificial Systems.

[5] Price GR (1970). Selection and covariance.. Nature.

[6] Frank SA (1997). Developmental selection and self-organization.. BioSystems.

[7] Frank SA (1997). The design of adaptive systems: optimal parameters for variation and selection in learning and development.. Journal of Theoretical Biology.

[8] Izhikevich EM (2006). Polychronization: computation with spikes.. Neural Computation.

[9] Harvey I, Kampis G (2011). The Microbial Genetic Algorithm.. ECAL 2009.

[10] Toussaint M (2003). The evolution of genetic representations and modular adaptation. ND 04, 44780.

[11] Vasas V, Szathmáry E, Santos M (2010). Lack of evolvability in self-sustaining autocatalytic networks constraints metabolism-first scenarios for the origin of life.. Proc Natl Acad Sci U S A.

[12] Adams P (1998). Hebb and Darwin.. J Theor Biol.

[13] Chklovskii DB, Mel BW, Svoboda K (2004). Cortical rewiring and information storage.. Nature.

[14] Butz M, Worgotter F, van Ooyen A (2009). Activity-dependent structural plasticity.. Brain Research Reviews.

[15] Chu PC, Beasley JE (1998). A Genetic Algorithm for the Multidimensional Knapsack Problem.. Journal of Heuristics.

[16] Khuri S, Back T, Heitkotter J, Deaton E (1994). The zero/one multiple knapsack problem and genetic algorithms.. Proc 1994 ACM Symp Applied Computing.

[17] Kirchner M, Gerhart J (1998). Evolvability.. Proc Natl Acad Sci USA.

[18] Pigliucci M (2008). Is evolvability evolvable?. Nature Reviews Genetics.

[19] Kashtan N, Alon U (2005). Spontaneous evolution of modularity and network motifs.. Proc Natl Acad Sci USA.

[20] Izquierdo E, Fernando C, Bullock S, Noble J, Watson RA, Bedau MA (2008). The evolution of evolvability in gene transcription networks.. Proceedings of the 11th International Conference on Artificial Life.

[21] Watson RA, Hornby GS, Pollack JB (1998). Modelling Building-Block Interdependency..

[22] Watson RA, Buckley CL, Mills R (2009). The Effect of Hebbian Learning on Optimisation in Hopfield Networks..

[23] Watson RA (2006). Compositional Evolution: The Impact of Sex, Symbiosis, and Modularity on the Gradualist Framework of Evolution.

[24] Fernando C, Goldstein R, Szathmáry E (2010). The Neuronal Replicator Hypothesis.. Neural Computation.

[25] Fernando C, Karishma KK, Szathmáry E (2008). Copying and Evolution of Neuronal Topology.. PLoS ONE.

[26] Fernando C, Szathmáry E, Glatzeder B, Goel V, von Müller A (2009). Natural selection in the brain.. Toward a Theory of Thinking.

[27] Fernando C, Szathmáry E, Pigliucci M, Müller G (2009). Chemical, neuronal and linguistic replicators.. Towards an Extended Evolutionary Synthesis.

[28] Fernando C (2010). Neuronal Replication Solves the Stability-Plasticity Dilemma..

[29] Fernando C (2011). Symbol Manipulation and Rule Learning in Spiking Neuronal Networks.. Journal of Theoretical Biology.

[30] Maynard-Smith J, Nitecki MH (1988). Evolutionary progress and the levels of selection.. Evolutionary Progress.

[31] Masquelier T, Guyonneau R, Thorpe SJ (2009). Competitive STDP-Based Spike Pattern Learning.. Neural Computation.

[32] Song S, Miller KD, Abbott L (2000). Competitive Hebbian Learning Through Spike-Timing Dependent Synaptic Plasticity.. Nature Neurosci.

[33] Hebb DO (1949). The Organization of Behaviour: A Neuropsychological Theory.

[34] Sutton SR, Barto AG (1998). Reinforcement Learning: An Introduction.

[35] Cisek P (2006). Integrated Neural Processes for Defining Potential Actions and Deciding between Them: A Computational Model.. The Journal of Neuroscience.

[36] Cisek P, Kalaska JF (2005). Neural correlates of reaching decisions in dorsal premotor cortex: specification of multiple direction choices and final selection of action.. Neuron.

[37] Desimone R, Duncan J (1995). Neural Mechanisms of Selective Visual Attention.. Annu Rev Neurosci.

[38] Pelikan M, Goldberg DE, Cabt-paz E (2000). Linkage Problem, Distribution Estimation and Bayesian Networks.. Evolutionary Computation.

[39] Dorigo M, Stutzle T (2004). Ant Colony Optimization.

[40] Dorigo M, Blum C (2005). Ant colony optimization theory: A survey.. Theoretical Computer Science.

[41] Fontana W, Buss LW (1994). What would be conserved if ‘the tape were played twice’?. Proc Natl Acad Sci USA.

[42] Poli R, Kennedy J, Blackwell T (2007). Particle Swarm Optimization: An overview.. Swarm Intelligence.

[43] Eddy SR (1996). Hidden Markov Models.. Current Opinion in Structural Biology.

[44] Baum LE, Petrie T, Soules G, Weiss N (1970). A maximization technique occurring in the statistical analysis of probabilistic functions of Markov chains.. Ann Math Statist.

[45] Thomsen R (2002). Evolving the Topology of Hidden Markov Models using Evolutionary Algorithms..

[46] Schwarz GE (1978). Estimating the dimension of a model.. Annals of Statistics.

[47] Izhikevich EM (2007). Solving the Distal Reward Problem through Linkage of STDP and Dopamine Signaling.. Cerebral Cortex.

[48] Holtmaat A, Sovoboda K (2009). Experience-dependent structural plasticity in the mammalian brain.. Nature Reviews Neurosicence.

[49] Lohmann C, Bonhoeffer T (2008). A Role for Local Calcium Signaling in Rapid Synaptic Partner Selection by Dendritic Filopodia.. Neuron.

[50] Schmidhuber J (2000). Evolutionary Computation versus Reinforcement Learning..

[51] Doya K, Samejima K, Katagiri K, Kawato M (2002). Multiple Model-Based Reinforcement Learning.. Neural Computation.

[52] Izhikevich EM, Hoppensteadt FC (2009). Polychronous Wavefront Computations.. International Journal of Bifurcation and Chaos.

[53] Butz AMV, Goldberg BDE, Stolzmann CW (2002). The anticipatory classifier system and genetic generalization..

